# Comparative effectiveness of metronidazole and vancomycin for treatment of *Clostridioides difficile* infection in hospitalized children

**DOI:** 10.1017/ash.2025.51

**Published:** 2025-03-12

**Authors:** Thomas J. Sandora, Timothy J. Savage, Morgan E. Ryan, Suzanne E. Dahlberg, Kaitlyn Daugherty, Ciarán P. Kelly, Nira R. Pollock, Larry K. Kociolek

**Affiliations:** 1 Division of Infectious Diseases, Department of Pediatrics, Boston Children’s Hospital and Harvard Medical School, Boston, MA, USA; 2 Division of Pharmacoepidemiology and Pharmacoeconomics, Department of Medicine, Brigham and Women’s Hospital and Harvard Medical School, Boston, MA, USA; 3 Biostatistics Research and Design Center, Boston Children’s Hospital, Boston, MA, USA; 4 Division of Pulmonary Medicine, Department of Pediatrics, Boston Children’s Hospital and Harvard Medical School, Boston, MA, USA; 5 Division of Gastroenterology (K.D., C.K) and Division of Infectious Diseases (N.R.P.), Department of Medicine, Beth Israel Deaconess Medical Center and Harvard Medical School, Boston, MA, USA; 6 Department of Laboratory Medicine, Boston Children’s Hospital and Harvard Medical School, Boston, MA, USA; 7 Division of Infectious Diseases, Department of Pediatrics, Ann & Robert H. Lurie Children’s Hospital of Chicago and Northwestern University Feinberg School of Medicine, Chicago, IL, USA

## Abstract

**Objective:**

To compare rates of clinical response in children with *Clostridioides difficile* infection (CDI) treated with metronidazole vs vancomycin.

**Design:**

Retrospective cohort study was performed as a secondary analysis of a previously established prospective cohort of hospitalized children with CDI. For 187 participants 2–17 years of age who were treated with metronidazole and/or vancomycin, the primary outcome of clinical response (defined as resolution of diarrhea within 5 days of treatment initiation) was identified retrospectively. Baseline variables associated with the primary outcome were included in a logistic regression propensity score model estimating the likelihood of receiving metronidazole vs vancomycin. Logistic regression using inverse probability of treatment weighting (IPTW) was used to estimate the effect of treatment on clinical response.

**Results:**

One hundred seven subjects received metronidazole and 80 subjects received vancomycin as primary treatment. There was no univariable association between treatment group and clinical response; 78.30% (N = 83) of the metronidazole treatment group and 78.75% (N = 63) of the vancomycin group achieved clinical response (*P* = 0.941). After adjustment using propensity scores with IPTW, the odds of a clinical response for participants who received metronidazole was 0.554 (95% CI: 0.272, 1.131) times the odds of those who received vancomycin (*P* = 0.105).

**Conclusions:**

In this observational cohort study of pediatric inpatients with CDI, the rate of resolution of diarrhea after 5 days of treatment did not differ among children who received metronidazole vs vancomycin.

## Introduction

There are limited data evaluating comparative effectiveness of antibiotics for treatment of *Clostridioides difficile* infection (CDI) in children. Adult data from randomized controlled trials demonstrated that metronidazole is inferior to vancomycin,^1^ resulting in a change in the 2017 Infectious Diseases Society of America (IDSA) and Society for Healthcare Epidemiology of America (SHEA) CDI treatment guidelines^2^ (and reinforced in 2021^3^) in which metronidazole was removed as first-line treatment for CDI in adults. Metronidazole was recommended for non-severe CDI only if both fidaxomicin and vancomycin were unavailable. However, because of limited pediatric data, the 2017 IDSA/SHEA guidelines recommend either metronidazole or vancomycin as acceptable options for a first episode of non-severe pediatric CDI.

Metronidazole has never been evaluated in a randomized controlled trial for treatment of pediatric CDI. A retrospective observational single-center cohort study of hospitalized children with CDI found that vancomycin may be associated with earlier symptom resolution compared with metronidazole, but the authors suggested additional studies to demonstrate the reproducibility of this finding (because of limitations such as potential misclassification of outcome) and to verify generalizability to regions with different clinical practices and where the epidemiology of *C. difficile* ribotypes may differ.^4^ A subsequent systematic review and meta-analysis of pediatric patients found no significant difference in clinical cure rates between metronidazole and vancomycin, but geographic subgroup analyses demonstrated a significantly lower cure rate for metronidazole in the United States and Europe.^5^ However, only observational studies were included, and there was no assessment of control for confounding in the included studies.

Given a continued lack of clarity about the role of metronidazole as a treatment option for CDI in children, we used a pre-existing cohort of hospitalized children with CDI to compare the effectiveness of metronidazole and vancomycin in achieving clinical response by day 5 of treatment. Our hypothesis was that the clinical response rate would be lower in children treated with metronidazole.

## Methods

### Study design

We examined data from a National Institutes of Health (NIH)-funded prospective cohort study of stool toxin concentrations in pediatric CDI.^6^ For the original cohort, eligible inpatients at Boston Children’s Hospital (BCH, Boston, MA) and Ann & Robert H. Lurie Children’s Hospital of Chicago (Chicago, IL) were prospectively enrolled between July 13, 2016 and April 4, 2019 under protocols approved by the Institutional Review Boards at each institution. Subjects were ≤ 17 years old with positive stool *C. difficile* glutamate dehydrogenase/toxin or nucleic acid amplification testing confirmatory test, initiated CDI therapy, and had acute diarrhea, defined as: a) ≥ 3 unformed bowel movements during any 24 hours in the 48 hours before or the 24 hours after the time of stool collection; OR b) persistent diarrhea in the same time window, per multiple provider notes; OR c) pseudomembranous colitis; OR d) in patients with chronic diarrhea, a clear change in stool consistency or frequency. In most cases, definition “a” was applied. Assessment for the presence of diarrhea included review of nursing logs for number and consistency of stools and detailed chart review. Whether a patient met the diarrhea criteria was adjudicated for all patients by one of three clinical research assistants, who conferred to reach consensus about questions and who called the patient’s nurse and/or asked the parent/guardian about diarrhea if the notes suggested diarrhea but were not clear enough to be sure. Subjects were excluded if they had chronic diarrhea without clear exacerbation, a diagnostic specimen of insufficient volume or >72 hours old, received CDI treatment for >48 hours prior to stool collection or had a colostomy. Peak white blood cell (WBC) count and creatinine values within 5 days before to 2 days after stool collection were recorded.

Patients were followed for 40 days from study enrollment (starting from the date of diagnostic stool sample) via chart review or by phone call. The duration of CDI treatment was measured as the number of days on treatment for CDI from initiation to the first discontinuation of treatment. The study team assigned baseline CDI severity using IDSA-SHEA criteria (severe CDI was defined as WBC count ≥15,000/µl and/or creatinine level ≥1.5 g/dl).^2^

For the current treatment analysis, subjects were excluded if they were younger than 2 years of age (because of high colonization rates in this age group resulting in lack of certainty that a positive test reflects CDI rather than colonization). The primary outcome was documented clinical response (defined as resolution of diarrhea within 5 days of antibiotic treatment initiation, with the first calendar day of treatment defined as day 1) and was identified retrospectively by chart review by two authors (TJSandora and LKK). The primary exposure of interest was receipt of vancomycin or metronidazole. Children who received vancomycin on at least 3 consecutive calendar days within the first 5 days of CDI therapy were assigned to the vancomycin group (regardless of whether metronidazole was concurrently administered), and children who received vancomycin on only 1–2 calendar days within the first 5 days of CDI therapy but completed the first 5 days of treatment with metronidazole were assigned to the metronidazole group. A sensitivity analysis was performed excluding patients who received both metronidazole and vancomycin during the first 5 days of treatment.

### Statistical analysis

Descriptive statistics were used to summarize baseline patient demographics and clinical characteristics overall, by treatment group, and by clinical response. Fisher’s exact test for categorical variables and Wilcoxon rank sum test for continuous variables were used to test for differences between groups. The proportions of patients who achieved clinical response in each treatment group were compared using a χ^2^ test.

To adjust for potential confounding, we estimated propensity scores using logistic regression and then applied inverse probability treatment weighting (IPTW). Simulation studies have shown that variables associated with the outcome of interest should be included in the estimation of propensity score regardless of their associations with the treatment. Including variables that are associated with the treatment but not the outcome can decrease precision without improving bias.^7^ Therefore, baseline variables that were significantly associated with the primary outcome of clinical response at *P* ≤ .2 were included in a logistic regression propensity score model. A priori theorized confounders including treatment start year, race/ethnicity, and past treatment of CDI were also included in the propensity score model; race/ethnicity has been associated with both antibiotic treatment and CDI outcomes in prior studies.^8,9^ We dichotomized treatment start year as 2016/2017 (before IDSA-SHEA guideline publication) and 2018/2019 (after guideline publication). Average treatment effect weights were then stabilized and balance of baseline covariates between treatment groups was assessed, with standardized differences within ±0.25 and variance ratios between 0.5 and 2 indicating successful balance for a particular covariate.^10^ Variables with a standardized difference larger than ±0.25 were evaluated as covariates in adjusted models to control for residual confounding but were removed if they had a *P* value greater than 0.2. An interaction term for treatment start year*treatment group was evaluated for inclusion in the model to account for any secular changes. Weighted logistic regression using the IPTW weights was used to estimate the effect of treatment on the outcome of clinical response.

Given a fixed sample size of 106 patients receiving metronidazole and 80 patients receiving vancomycin, and an expected clinical response rate to metronidazole of 75%,^4^ we estimated we would have 80% power to detect a 15% increase in clinical response in the vancomycin group using a two-sided, two-sample *Z*-Test for a difference in proportions with unpooled variance, with a Type I error rate (α) of 0.05.

Analyses were conducted using SAS version 9.4 (SAS Institute, Cary, NC). All tests were two-tailed, and alpha was set at 0.05.

## Results

Two hundred nine subjects were enrolled in the original study. One subject was excluded for having a primary treatment (rifaximin) other than metronidazole or vancomycin. An additional 21 subjects were excluded because they were younger than 2 years of age. Table 1 shows baseline demographics and clinical characteristics overall and by treatment group for 187 subjects. One hundred seven subjects received metronidazole and 80 received vancomycin as primary treatment. At baseline, patients in Boston were significantly more likely to receive vancomycin than those in Chicago, as were those with inflammatory bowel disease, a prior CDI diagnosis, and those treated in 2018–2019 (ie, after the 2017 IDSA/SHEA guideline was published). Within 40 days after study enrollment, 38 patients (20.32%) were admitted to the intensive care unit, 2 (1.07%) had a colectomy, and 3 (1.60%) died. Seventeen patients (9.09%) experienced CDI recurrence within 40 days of enrollment (12/107 [11.21%] in the metronidazole group and 5/80 [6.25%] in the vancomycin group).

**Table 1.** Baseline demographics and clinical characteristics overall and by treatment group (N = 187)

		Overall (N = 187)	Metronidazole (N = 107)	Vancomycin (N = 80)
Site	Boston (%)	90 (48.13)	27 (25.23)	63 (78.75)
	Chicago (%)	97 (51.87)	80 (74.77)	17 (21.25)
Age	Mean (SD)	8.86 (4.60)	10.15 (4.55)	9.47 (4.67)
	Median (Q1, Q3)	9.63 (5.80, 13.57)	10.22 (6.06, 13.62)	8.40 (5.62, 13.39)
	[Min, Max]	[2.04, 17.88]	[2.04, 17.88]	[2.11, 17.76]
Sex	Male (%)	108 (57.75)	65 (60.75)	43 (53.75)
	Female (%)	79 (42.25)	42 (39.25)	37 (46.25)
Race/Ethnicity	White Non-Hispanic (%)	88 (47.06)	55 (51.40)	33 (41.25)
	Black Non-Hispanic (%)	17 (9.09)	12 (11.21)	5 (6.25)
	Hispanic (%)	37 (19.79)	22 (20.56)	15 (18.75)
	Other (%)	45 (24.06)	18 (16.82)	27 (33.75)
Inflammatory bowel disease	Yes (%)	23 (12.30)	8 (7.48)	15 (18.75)
	No (%)	164 (87.70)	99 (92.52)	65 (81.25)
Receiving chemotherapy	Yes (%)	58 (31.02)	35 (32.71)	23 (28.75)
	No (%)	129 (68.98)	72 (67.29)	57 (71.25)
Hematopoietic stem cell transplant recipient	Yes (%)	24 (12.83)	13 (12.15)	11 (13.75)
	No (%)	163 (87.17)	94 (87.85)	69 (86.25)
Immunosuppression^a^ (N = 24)	Yes (%)	17 (20.83)	10 (76.92)	7 (63.64)
	No (%)	7 (29.17)	3 (23.08)	4 (36.36)
Graft vs host disease^a^ (N = 24)	Yes (%)	3 (12.50)	2 (15.38)	1 (9.09)
	No (%)	21 (87.50)	11 (84.62)	10 (90.91)
History of solid organ transplant	Yes (%)	21 (11.23)	8 (7.48)	13 (16.25)
	No (%)	166 (88.77)	99 (92.52)	67 (83.75)
Immunocompromised host^b^	Yes (%)	88 (47.06)	48 (44.86)	40 (50.00)
	No (%)	99 (52.94)	59 (55.14)	40 (50.00)
Past CDI diagnosis	Yes (%)	36 (19.25)	10 (9.35)	26 (32.50)
	No (%)	151 (80.75)	97 (90.65)	54 (67.50)
Time from last CDI diagnosis to treatment start (days) (N = 36)	Mean (SD)	284.55 (497.90)	773.56 (728.39)	84.50 (87.34)
	Median (Q1, Q3)	82.00 (26.00, 288.00)	340.00 (288.00, 1172.00)	60.00 (24.00, 105.00)
	[Min, Max]	[14.00, 2166.00]	[63.00, 2166.00]	[14.00, 376.00]
	Freq. of Missing	5	1	4
IDSA-SHEA severity	Not Severe (%)	122 (65.24)	65 (60.75)	57 (71.25)
	Severe (%)	34 (18.18)	19 (17.76)	15 (18.75)
	Freq. of Missing	31	23	8
Treatment start year	2016/2017 (%)	129 (68.98)	97 (90.65)	32 (40.00)
	2018/2019 (%)	58 (31.02)	10 (9.35)	48 (60.00)

CDI, *C. difficile* infection; IDSA, Infectious Diseases Society of America; SHEA, Society for Healthcare Epidemiology of America.

a Immunosuppression (defined as receiving immunosuppressive agents) and graft vs host disease were assessed at the time of CDI diagnosis and only among hematopoietic stem cell transplant recipients.

b Immunocompromised host was defined as receiving chemotherapy and/or prior hematopoietic stem cell or solid organ transplant.

**Figure 1.**
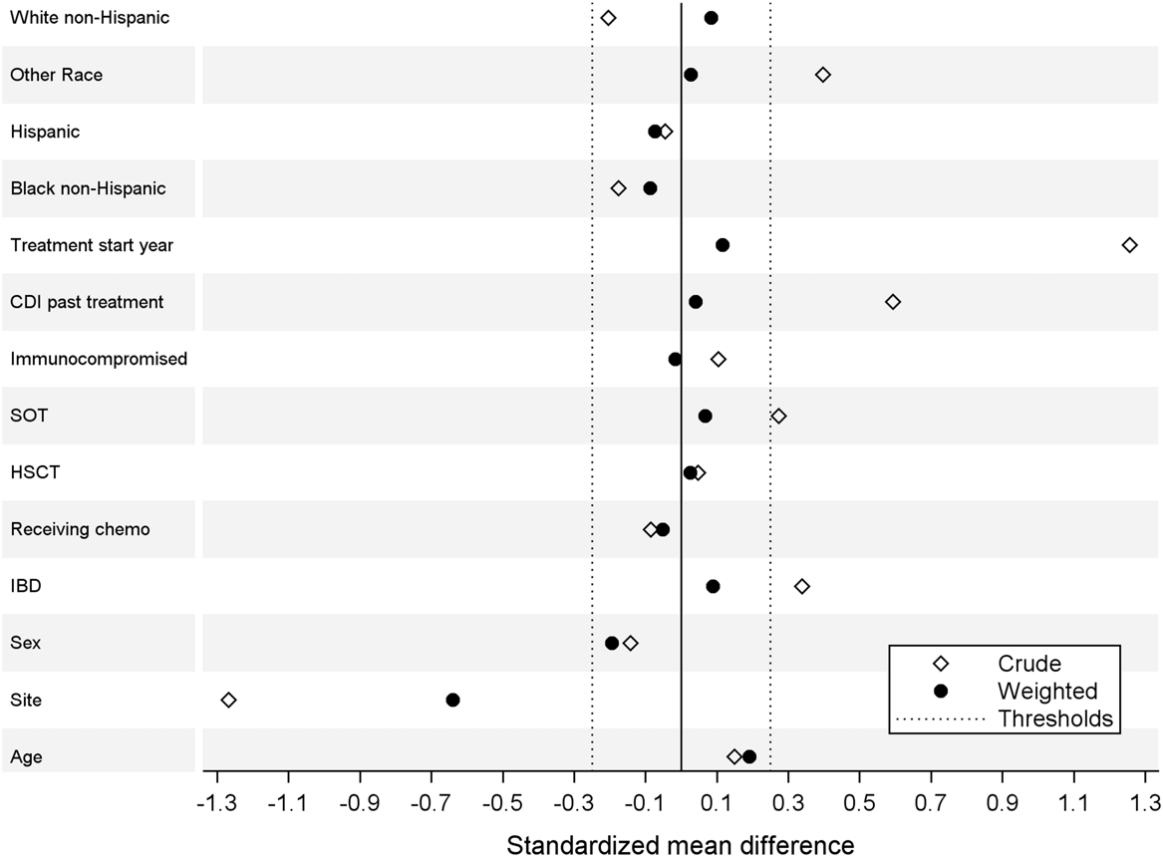
Standardized mean differences for all baseline variables. All of the weighted observations between the vertical threshold lines are less than 0.25 in absolute value, indicating that the variable is in balance after weighting. CDI, *C. difficile* infection; SOT, solid organ transplantation; HSCT, hematopoietic stem cell transplantation; chemo, chemotherapy; IBD, inflammatory bowel disease.

One subject who received metronidazole had missing data about clinical response, leaving 186 patients for the analysis of the primary outcome. There was no univariable association between treatment group and the primary outcome, with 78.30% (n = 83) of the metronidazole group and 78.75% (n = 63) of the vancomycin group achieving clinical response within 5 days of treatment initiation (unadjusted odds ratio 0.974 [95% CI 0.480, 1.975], *P* = .941). Table 2 displays baseline demographics and clinical characteristics by the outcome of clinical response. In univariable analysis, receiving chemotherapy and being a hematopoietic stem cell transplant recipient were each associated with a significantly higher likelihood of clinical response. Baseline CDI severity was not significantly associated with clinical response. Sex, inflammatory bowel disease, receipt of chemotherapy, receipt of solid organ transplant, receipt of hematopoietic stem cell transplant, and immunocompromised status were associated with clinical response at *P* ≤ .2 and included in the logistic regression propensity score model (in addition to treatment start year, race/ethnicity, and past treatment of CDI as described above). The standardized mean differences shown in Figure 1 and Supplemental Table 1, as well as the variance ratios provided in Supplemental Table 1, indicated covariate balance before and after weighting; the only variable that remained imbalanced between treatment groups after weighting was site (Boston or Chicago). Supplemental Figure 1 displays the propensity score distribution before and after weighting among participants in the metronidazole and vancomycin treatment groups. Neither site nor the interaction effect between treatment start year and treatment group were significant when added to a multivariable model and therefore neither was included in the final model reported. Table 3 displays the results of the final stabilized IPTW logistic regression model; the odds of a clinical response among patients receiving metronidazole compared with vancomycin was 0.554 (95% CI: 0.272, 1.131) (*P* = .105).

**Table 2.** Baseline demographics and clinical characteristics by clinical response (N = 186)

		Clinical response (N = 146)	No clinical response (N = 40)	*P*-value^a^
Site	Boston (%)	73 (50.00)	17 (42.50)	.476
	Chicago (%)	73 (50.00)	23 (57.50)	
Age	Mean (SD)	9.89 (4.45)	9.76 (5.21)	.846
	Median (Q1, Q3)	9.60 (6.06, 13.46)	9.55 (4.83, 15.28)	
	[Min, Max]	[2.08, 17.88]	[2.04, 17.58]	
Sex	Male (%)	88 (60.27)	19 (47.50)	**.154**
	Female (%)	58 (39.73)	21 (52.50)	
Race/Ethnicity	White Non-Hispanic (%)	68 (46.58)	20 (50.00)	.979
	Black Non-Hispanic (%)	14 (9.59)	3 (7.50)	
	Hispanic (%)	28 (19.18)	8 (20.00)	
	Other (%)	36 (24.66)	9 (22.50)	
Inflammatory bowel disease	Yes (%)	14 (9.59)	9 (22.50)	**.053**
	No (%)	132 (90.41)	31 (77.50)	
Receiving chemotherapy	Yes (%)	51 (34.93)	7 (17.50)	**.036**
	No (%)	95 (65.07)	33 (82.50)	
Hematopoietic stem cell transplant recipient	Yes (%)	23 (15.75)	1 (2.50)	**.031**
	No (%)	123 (84.25)	39 (97.50)	
Immunosuppression (N = 24)	Yes (%)	16 (69.57)	1 (100.00)	1.000
	No (%)	7 (30.43)	0 (0.00)	
Graft vs host disease (N = 24)	Yes (%)	3 (13.04)	0 (0.0)	1.000
	No (%)	20 (86.96)	1 (100.00)	
History of solid organ transplant	Yes (%)	14 (9.59)	7 (17.50)	**.167**
	No (%)	132 (90.41)	33 (82.50)	
Immunocompromised host	Yes (%)	74 (50.68)	14 (35.00)	**.107**
	No (%)	72 (49.32)	26 (65.00)	
Past CDI diagnosis	Yes (%)	26 (17.81)	10 (25.00)	.366
	No (%)	120 (82.19)	30 (75.00)	
Time from last CDI diagnosis to treatment start (days) (N = 36)	Mean (SD)	209.41 (381.83)	468.22 (702.47)	.396
	Median (Q1, Q3)	79.50 (27.00, 156.00)	288.00 (25.00, 376.00)	
	[Min, Max]	[14.00, 1530.00]	[17.00, 2166.00]	
	Freq. of Missing	4	1	
IDSA-SHEA severity	Not Severe (%)	99 (67.81)	22 (55.00)	.447
	Severe (%)	30 (20.55)	4 (10.00)	
	Freq. of Missing	17	14	
Treatment start year	2016/2017 (%)	97 (66.44)	31 (77.50)	.248
	2018/2019 (%)	49 (33.56)	9 (22.50)	

CDI, *C. difficile* infection; IDSA, Infectious Diseases Society of America; SHEA, Society for Healthcare Epidemiology of America.

a Fisher’s exact test for categorical variables and Wilcoxon rank sum test for continuous variables. Bolded *P*-values are *P* ≤ .2 and were included in the propensity score model.

**Table 3.** Unadjusted and adjusted odds of clinical response to metronidazole compared with vancomycin (N = 186)

Primary treatment group	Clinical response (%)	Unadjusted odds ratio (95% CI)	Adjusted^a^ odds ratio (95% CI)
Metronidazole (n = 106)	83/106 (78.30%)	0.974 (0.480, 1.975)	0.554 (0.272, 1.131)
Vancomycin (n = 80)	63/80 (78.75%)	Reference	Reference

CI, confidence interval.

a Weighted logistic regression model using inverse probability of treatment weights; treatment group is the only independent variable.

Twenty-five subjects who received at least one dose of both vancomycin and metronidazole during the first 5 days of treatment were excluded from a sensitivity analysis. Of the remaining 161 subjects, 98 (60.87%) received metronidazole and 125 (77.64%) had a clinical response. There continued to be no crude association between treatment and clinical response (unadjusted odds ratio 1.146 [95% CI 0.539, 2.436], *P* = .723). In the stabilized IPTW logistic regression model including adjustment for age (which was imbalanced between groups among this subset of patients), the odds of a clinical response among those who received metronidazole was 0.999 (95% CI: 0.459, 2.177) (*P* = .999) times those that received vancomycin.

## Discussion

In this study of hospitalized children with CDI using propensity scores to account for confounding, resolution of diarrhea within 5 days of treatment initiation did not differ significantly between the metronidazole and vancomycin treatment groups. The true estimate of effect from our model could fall anywhere within the observed confidence interval, which includes values on both sides of the null. There is no prospective comparative effectiveness study of these agents in children with CDI, making it difficult for clinicians to know whether there is still a role for metronidazole as a treatment option. While a meta-analysis found a lower odds of clinical cure among children treated with metronidazole versus vancomycin in the U.S. and Europe, this analysis included four studies; three were small and the fourth received a weight of 83% in the meta-analysis. The fourth study of a total of 192 patients found a lower odds of clinical improvement among patients treated with metronidazole (OR 0.40, 95% CI 0.17–0.97). The current study adds to the literature through comparison of these antibiotics in geographically distinct sites and in a more diverse population.

The IDSA/SHEA guidelines do not express a preference for metronidazole or vancomycin for mild to moderate pediatric CDI, in part because of the historical experience of successful use of metronidazole in this context. Although the use of vancomycin to treat pediatric CDI has increased over time,^8^ a 2014 survey of pediatric infectious diseases physicians found that they overwhelmingly used metronidazole for a first episode of non-severe disease.^11^

If newer data demonstrated superiority of vancomycin, they could be the basis for a new recommendation in subsequent guideline updates. There are several plausible explanations for why our study did not demonstrate a difference between these antibiotics. A negative result can be related to insufficient power; we had adequate power to identify a 15% difference favoring vancomycin, based on published pediatric data.^4^ In adult studies the difference in response rate between these agents was <10%.^1^ Our results suggest that if there is a difference between these treatments in children, it may be of similar magnitude or smaller than what has been observed in adults, which in part could explain why response rates to metronidazole for pediatric CDI historically have been high.^2,12^ It is also possible that this cohort of patients had milder CDI and that metronidazole may not differ substantially from vancomycin in this context. Although 18% of children in the present study met the IDSA/SHEA criteria for severe disease, we may not have reliably captured CDI severity because those criteria have been shown to poorly discriminate between children with and without severe disease.^13^

Another possibility is that we might have identified a significant difference between groups if we assessed the outcome at a later point in time. We chose day 5 because a prior retrospective study demonstrated improved resolution of diarrhea at that timepoint among children who received vancomycin.^4^ However, a group of experts recently advocated for a revised definition of clinical endpoints in CDI treatment trials, suggesting that initial response at end of treatment should be assessed by day 2 after completion of primary CDI therapy (ie, assessed on days 11 and 12 of a 10-day treatment course).^14^ The primary benefit of this approach is that it would increase the number of trial participants who are eligible to achieve sustained clinical response (a more holistic and important outcome for patients with CDI). There is a newer recommendation to use fidaxomicin to treat adults with CDI, because of its lower recurrence rate compared with vancomycin (a benefit that has also been demonstrated prospectively in children).^3,15^ Future studies could evaluate success at day 11–12 or time to clinical response in pediatric cohorts.

Although a meta-analysis of pediatric CDI treatment found that vancomycin was superior to metronidazole in the U.S. and Europe,^5^ it was subject to limitations that raise uncertainty about the conclusion. Only one of the seven included studies were performed outside the U.S. and Europe, and outcome definitions within individual studies varied (eg, “resolving CDI symptoms,” “resolution or improvement of diarrhea within 5 days,” “cure,” “treatment failure”). The clinical cure rates when all 7 studies were included were not significantly different between groups. The authors suggested that the difference seen in subgroup analysis was related to the higher prevalence of ribotype 027 in the U.S. and Europe, but molecular studies of strains from children with CDI in the U.S. have found that ribotype 027 accounts for a small proportion of cases.^16,17^

Even if there is no difference in the effectiveness of these agents for treating pediatric CDI, there are other factors to consider. Antimicrobial stewardship includes optimizing antibiotic selection not only to maximize treatment outcomes but also to minimize consequences such as adverse drug events and the emergence of resistance.^18^ Although oral vancomycin is not absorbed to an appreciable extent, a prior meta-analysis demonstrated no significant differences in adverse event rates between metronidazole and vancomycin in patients with CDI.^19^ In mouse models investigators have demonstrated a greater loss of microbiota-mediated colonization resistance to *C. difficile* following oral vancomycin as compared with metronidazole, which can predispose to colonization by other antibiotic-resistant healthcare-associated pathogens.^20^ An emergence of circulating clinical *C. difficile* strains with reduced susceptibility to vancomycin has been documented,^21^ although the clinical significance of this finding is debatable since colonic concentrations of vancomycin typically substantially exceed minimum inhibitory concentrations of strains with reduced susceptibility. In contrast, metronidazole resistance has been increasing over time; in one study 18% of clinical *C. difficile* isolates from adult patients had a minimum inhibitory concentration of ≥1 µg/ml, which was an independent predictor of clinical treatment failure.^22^ Decisions about treatment choice must take into consideration the balance of potential benefits and harms.

Our study has several strengths, including the use of a prospectively-established cohort with strict definitions of diarrhea and the exclusion of children under the age of 2 years, increasing the confidence in the diagnosis of CDI, and the use of a propensity score analysis to account for potential confounding (eg, patients with more significant diarrheal symptoms and/or medical complexity may have been both more likely to receive vancomycin and less likely to respond quickly). This study also has at least 5 limitations. First, propensity scoring cannot entirely obviate the possibility of residual confounding. However, it is considered the preferred methodological approach for controlling confounding when a randomized trial is not feasible or available. Second, because we retrospectively assessed diarrhea resolution on day 5 for this analysis, misclassification of outcome based on inadequate or inaccurate documentation of diarrhea in the medical record is possible. While lack of blinding during chart review to the treatment received could theoretically introduce measurement bias, such an impact likely would have been in the opposite direction (eg, finding a significant difference between groups) from what our study showed. Third, site of treatment remained imbalanced between groups even after propensity score weighting, but adjusting for this covariate in a multivariable model did not change the result. Fourth, because these patients were hospitalized, the results may not be generalizable to treatment of pediatric CDI in the outpatient setting. Fifth, there may have been patients in both treatment groups who were asymptomatically colonized with *C. difficile* and had diarrhea from another unidentified cause.

Our data do not provide definitive evidence that metronidazole should be abandoned as an option for treatment of pediatric CDI. Future research should assess comparative effectiveness of various treatment strategies among children with non-severe CDI who do not require hospitalization.

## Supplementary material

The supplementary material for this article can be found at https://doi.org/10.1017/ash.2025.51

## Financial support

This work was supported by grants from the National Institutes of Health, National Institute of Allergy and Infectious Diseases [grant number 5 R01 AI116596 to N.R.P. and C.P.K.; K23 AI123525 to L.K.K.]. T.J. Savage was supported by the Eunice Kennedy Shriver National Institute of Child Health & Human Development of the NIH (Bethesda, MD) under award numbers T32HD040128 and K08HD110600. The content is solely the responsibility of the authors and does not necessarily represent the official views of the National Institutes of Health.

## Competing interests

L.K.K. receives grant support from Merck, unrelated to this study. C.P.K. has acted as a paid consultant to Artugen, Facile Therapeutics, Ferring, First Light Biosciences, Finch, Janssen (J&J), Matrivax, Merck, Seres, Pfizer and Vedanta. T.J. Savage received salary support via a contract to Brigham & Women’s Hospital by UCB. All other authors report no potential conflicts of interest.

## Supporting information

Sandora et al. supplementary material 1 Sandora et al. supplementary material

Sandora et al. supplementary material 2 Sandora et al. supplementary material
