# Supplementary figures and images for: Comparative effectiveness of metronidazole and vancomycin for treatment of Clostridioides difficile infection in hospitalized children
Source: Antimicrob Steward Healthc Epidemiol. 2025 Mar 12;5(1):e74. doi: 10.1017/ash.2025.51 (PMC11920915; doi:10.1017/ash.2025.51)

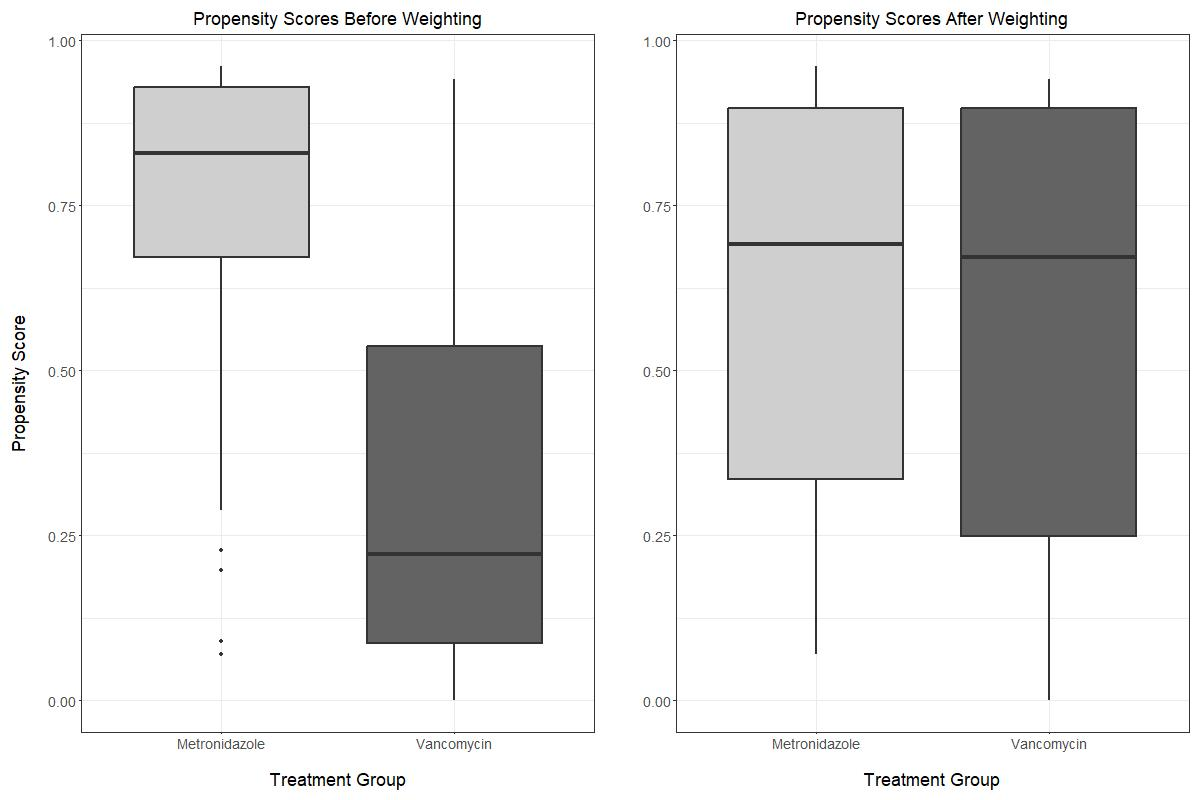

Supplement: Sandora et al. supplementary material 1 — Sandora et al. supplementary material [file S2732494X25000518sup001.tif]
