# Supplementary material for: Comparative effectiveness of metronidazole and vancomycin for treatment of Clostridioides difficile infection in hospitalized children
Source: Antimicrob Steward Healthc Epidemiol. 2025 Mar 12;5(1):e74. doi: 10.1017/ash.2025.51 (PMC11920915; doi:10.1017/ash.2025.51)
Supplement: Sandora et al. supplementary material 2 — Sandora et al. supplementary material [file S2732494X25000518sup002.docx]

Supplemental Table 1. Standardized Mean Differences and Variance Ratios Between Treatment Groups for Each Baseline Covariate Before and After Weighting

|  | **Before Weighting** | | **After Weighting** | |
| --- | --- | --- | --- | --- |
| **Variable** | **Mean Difference** | **Variance Ratio** | **Mean Difference** | **Variance Ratio** |
| Age | 0.148 | 0.951 | 0.191 | 1.041 |
| Site* | -1.268 | 1.127 | -0.639 | 1.016 |
| Sex | -0.142 | 0.959 | -0.194 | 0.976 |
| Inflammatory bowel disease | 0.339 | 0.454 | 0.088 | 0.786 |
| Receiving chemotherapy | -0.086 | 1.075 | -0.052 | 1.042 |
| Hematopoietic stem cell transplant recipient | 0.048 | 0.900 | 0.025 | 0.940 |
| History of solid organ transplant | 0.274 | 0.508 | 0.067 | 0.816 |
| Immunocompromised host | 0.103 | 0.989 | -0.016 | 1.004 |
| Past CDI treatment | 0.594 | 0.386 | 0.040 | 0.950 |
| Treatment start year | 1.257 | 0.353 | 0.116 | 0.907 |
| Black non-Hispanic | -0.177 | 1.699 | -0.086 | 1.371 |
| Hispanic | -0.046 | 1.072 | -0.074 | 1.133 |
| Other race/ethnicity | 0.397 | 0.626 | 0.028 | 0.960 |
| White non-Hispanic | -0.205 | 1.031 | 0.084 | 1.012 |

*Site of treatment (Boston Children’s Hospital or Lurie Children’s Hospital)

CDI, *C. difficile* infection
